# Neurological Manifestations in a Kidney Transplant Recipient: An Unexpected Case of Manganese Toxicity

**DOI:** 10.7759/cureus.108992

**Published:** 2026-05-16

**Authors:** Sakshi Vaishnav, Sherida Edding, Aurora Posadas, Santosh Nagaraju, Blaithin McMahon

**Affiliations:** 1 Department of Transplant Nephrology, Medical University of South Carolina, Charleston, USA; 2 Department of Transplant Surgery, Medical University of South Carolina, Charleston, USA; 3 Department of Nephrology, Medical University of South Carolina, Charleston, USA

**Keywords:** basal ganglia, chelation therapy, edta, heavy metal poisoning, kidney transplant, manganese toxicity, movement disorder, neurotoxicity, portal hypertension, tips

## Abstract

Manganese (Mn) toxicity is a relatively uncommon neurotoxic condition characterized by neuropsychiatric symptoms and parkinsonism. We describe a 46-year-old woman with end-stage renal disease due to congenital obstructive uropathy, status post deceased-donor kidney transplantation (2021), who presented with one month of worsening tremors, gait instability, intermittent aphasia, and altered awareness. Brain magnetic resonance imaging demonstrated persistent but improved T1 hyperintensity of the globus pallidus compared to prior imaging, consistent with Mn deposition. Serum Mn was markedly elevated at 14.9 µg/L (reference range 0.5-1.2 µg/L). Chelation therapy with calcium disodium ethylenediaminetetraacetic acid was initiated but discontinued early due to decline in renal function. Despite early cessation, treatment resulted in biochemical improvement and partial clinical recovery. Kidney allograft biopsy, performed due to a rise in creatinine during the hospital course, demonstrated severe chronic changes without evidence of acute rejection. Thorough history taking and evaluation of environmental factors did not identify a clear source of Mn exposure. The patient's history was notable for Budd-Chiari syndrome with portal hypertension status post transjugular intrahepatic portosystemic shunt, which likely contributed to impaired Mn clearance. To our knowledge, Mn toxicity occurring in a functioning kidney transplant recipient has not previously been reported. This case underscores the diagnostic and therapeutic challenges of Mn toxicity.

## Introduction

Manganese (Mn) is an essential trace element required for several metabolic and enzymatic processes, including neurotransmitter synthesis, mitochondrial function, and antioxidant defense through its role as a cofactor for Mn superoxide dismutase [1,2]. Normal brain Mn concentrations are tightly regulated, with the globus pallidus containing the highest physiologic levels [1]. At elevated concentrations, Mn crosses the blood-brain barrier and preferentially accumulates within the basal ganglia, particularly the globus pallidus. Deposition in these regions disrupts dopaminergic signaling pathways and produces the clinical syndrome of manganism [3,4].

Mn toxicity has been documented in several populations, including those with occupational exposure (welders, miners), patients receiving prolonged parenteral nutrition, individuals with chronic liver disease and portosystemic shunting, and patients on maintenance hemodialysis [5,6]. In patients with portosystemic shunts, including transjugular intrahepatic portosystemic shunts (TIPS), Mn accumulation results from bypass of hepatic first-pass metabolism and impaired hepatobiliary excretion [7,8]. Similarly, in hemodialysis patients, increased pallidal signal intensity has been observed despite circulating Mn levels comparable to controls, although the exact mechanism remains unclear [9]. However, Mn toxicity after kidney transplantation with a functioning renal allograft has not previously been reported.

## Case presentation

Patient background

We present the case of a 46-year-old woman with end-stage renal disease secondary to congenital obstructive uropathy, previously maintained on peritoneal dialysis, who underwent deceased-donor kidney transplantation in February 2021. Her medical history was notable for JAK2 V617F mutation-positive myelodysplastic/myeloproliferative neoplasm, diagnosed in 2014 following a bone marrow biopsy performed in the setting of recurrent left upper extremity ischemia requiring left upper extremity amputation (2013-2014). Additional history included hypertension, asthma, migraine, gastroesophageal reflux disease, gout, insomnia, anxiety, and peripheral neuropathy. Her posttransplant course was complicated by recurrent rejection episodes, and she remained on tacrolimus, mycophenolate, and prednisone with a baseline serum creatinine of 2.0-2.5 mg/dL.

One month following kidney transplantation (March 2021), she presented with abdominal distension, increased Jackson-Pratt drain output, and weight gain despite diuretics. Computed tomography of the abdomen and pelvis demonstrated splenomegaly, moderate-volume ascites, and findings suggestive of portal hypertension. She reported no prior history of liver disease and denied significant alcohol use. Liver biopsy revealed congestive hepatopathy with sinusoidal dilation and fibrosis. Hepatic venography demonstrated elevated free and wedged hepatic pressures (both 26 mmHg, gradient of 0), consistent with hepatic venous outflow obstruction. Gastroenterology concluded that she did not appear to have clinically significant intrinsic liver disease based on liver biopsy and negative work-up, and that her presentation was most consistent with Budd-Chiari physiology due to hepatic venous outflow obstruction, potentially related to ongoing thrombotic events in the setting of her underlying JAK2 mutation. TIPS was placed in April 2021. Esophagogastroduodenoscopy at that time revealed esophageal varices and portal hypertensive gastropathy.

Clinical findings

In July 2025, she presented to the emergency department with a one-month history of worsening tremors, gait imbalance, intermittent aphasia, and episodes of altered awareness. Detailed history obtained during subsequent neurology and toxicology evaluations revealed more gradual neuropsychiatric and cognitive symptoms beginning approximately 6-12 months prior to presentation, including intermittent auditory hallucinations, insomnia, mood changes, episodic confusion, memory impairment, and difficulty concentrating.

In the days preceding admission, she developed progressive motor neurologic symptoms, including involuntary jerking movements of the right upper extremity followed by bilateral lower extremity tremors and worsening gait instability. Additional symptoms included transient visual disturbances, persistent headache distinct from her prior migraines, and worsening word-finding difficulty over the preceding two weeks.

Neurologic examination demonstrated mixed extrapyramidal/parkinsonian and cerebellar findings, including increased tone in the right upper extremity with bradykinesia, a high-amplitude low-frequency tremor, dyssynergia on heel-to-shin testing bilaterally, and an impaired, wide-based gait with truncal ataxia. Reflexes were brisk, and a right Hoffman sign was present.

A detailed environmental, occupational, and dietary history did not identify a clear source of Mn exposure. The patient was not employed at the time of presentation and previously worked as an elementary school teacher and youth minister, with no history of occupational exposure to welding or industrial metals. Her diet consisted primarily of high-protein foods, including beef, chicken, and eggs, with limited intake of seafood, shellfish, nuts, or seeds. She denied significant intake of Mn-rich foods, including pumpkin seeds, sunflower seeds, and dark chocolate. She consumed municipal (city) water, which was found to have Mn levels well within the acceptable range as per the patient's report. She denied using herbal or alternative supplements. Her medications included over-the-counter vitamin C, magnesium, and vitamin D. Alcohol intake was minimal (one to two drinks per week), and she reported occasional marijuana use for insomnia. She denied use of illicit drugs, including cocaine, stimulants, or other substances. She lived with her husband and children, and there were no known environmental or household exposures to heavy metals.

Diagnostic assessment

Initial laboratory evaluation demonstrated stable baseline kidney allograft function with serum creatinine 2.5 mg/dL (baseline 2.0-2.5 mg/dL) and therapeutic tacrolimus levels. Electrolytes, acid-base status, calcium, magnesium, phosphorus, and liver function tests were largely unremarkable and did not suggest an alternative metabolic, infectious, or inflammatory etiology for her presentation.

**Figure 1:**
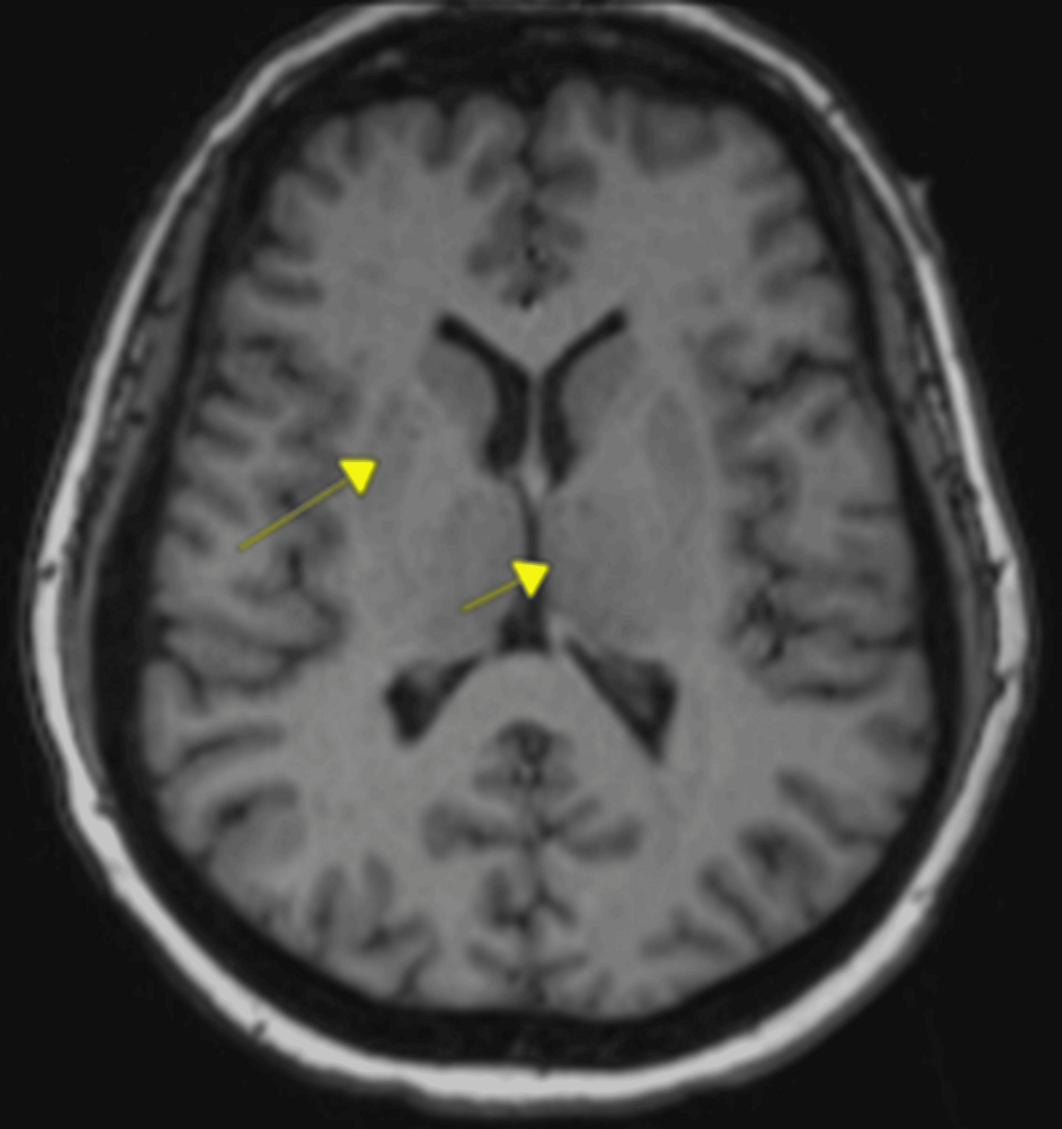
Axial T1-weighted MRI of the brain demonstrating bilateral symmetric hyperintensity within the globus pallidus (yellow arrows), consistent with manganese deposition MRI, magnetic resonance imaging

Computed tomography of the brain showed no acute infarction. Continuous electroencephalography demonstrated diffuse slowing without epileptiform activity, and cerebrospinal fluid analysis was negative for infectious or inflammatory etiologies. MRI demonstrated persistent but improved T1 hyperintensity of the globus pallidus compared to prior imaging (Figure 1), a characteristic imaging feature of Mn deposition [4,9]. Serum Mn was markedly elevated at 14.9 µg/L (reference range 0.5-1.2 µg/L).

Notably, a retrospective chart review revealed that a brain MRI obtained in 2023 for evaluation of memory decline and episodes concerning for seizures had also demonstrated "increased T1 signal intensity within the globus pallidus and anterior midbrain compatible with Mn-related MR signal changes from cirrhosis." Serum Mn levels do not appear to have been obtained at that time. The patient had been evaluated by neurology in December 2022 for "cognitive changes and episodes of loss of time" and had a subsequent follow-up in June 2023. This raises the possibility that she had ongoing Mn deposition about two years prior to her diagnosis.

Therapeutic intervention

**Figure 2:**
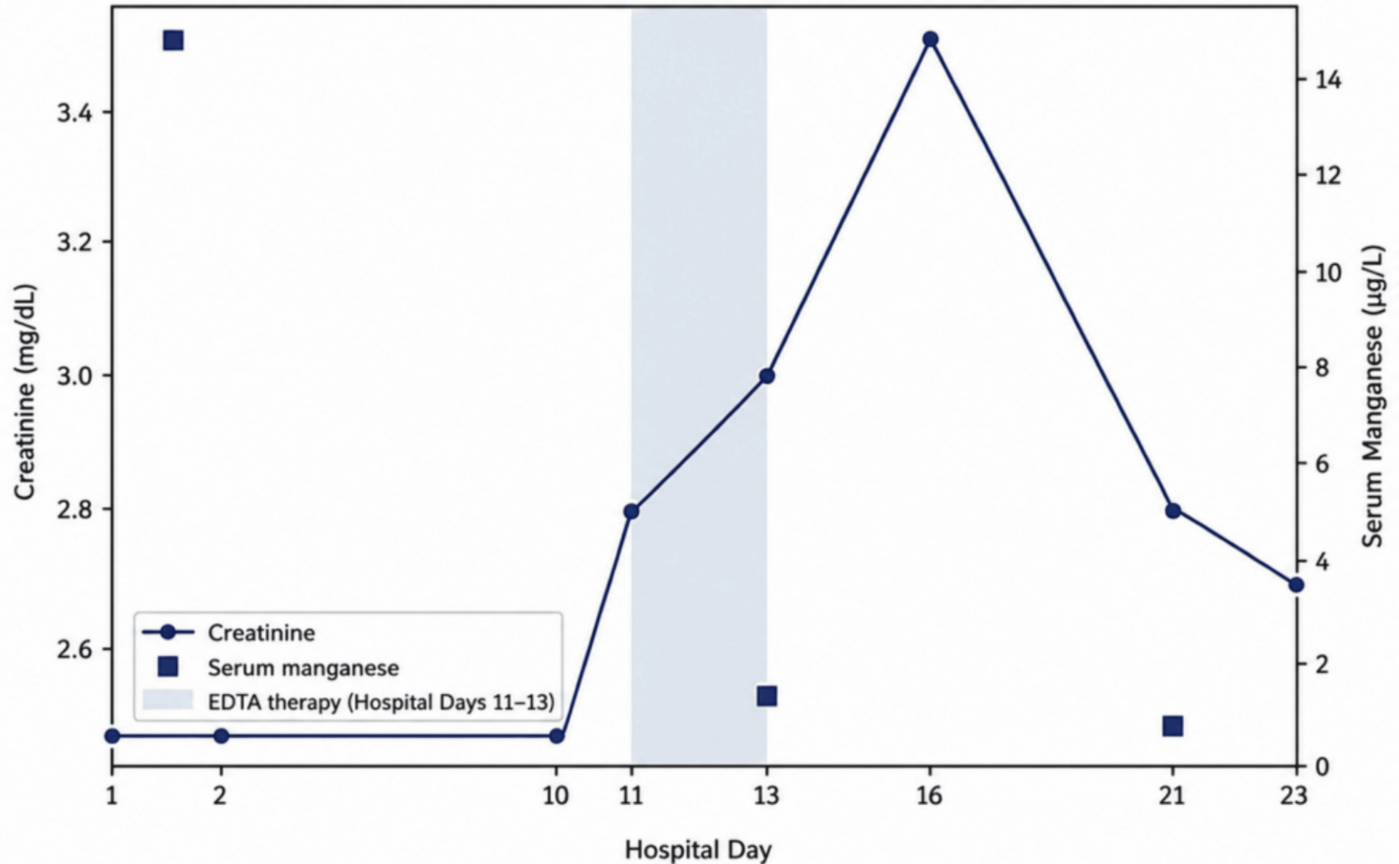
Trends in serum creatinine and manganese levels during hospitalization Serum manganese levels declined following initiation of calcium disodium EDTA therapy (hospital days 11-13). Serum creatinine increased during therapy and peaked several days after completion, followed by a downward trend prior to discharge. The shaded region represents the period of EDTA administration EDTA, ethylenediaminetetraacetic acid

Chelation therapy with calcium disodium ethylenediaminetetraacetic acid (EDTA) was initiated in early August 2025, given the patient's progressive neurologic manifestations, characteristic MRI findings, and markedly elevated serum Mn levels. Although nephrotoxicity is a recognized risk, therapy was pursued with careful monitoring of renal function to guide dose adjustments. Therapy was discontinued after three days due to an increase in serum creatinine (Table 1, Figure 2). Redosing was not required, as serum Mn levels and neurological symptoms subsequently improved.

**Table 1:** Selected laboratory trends during hospitalization Reference range for serum manganese: 0.5-1.2 µg/L. Serum manganese normalized following chelation therapy despite early discontinuation due to nephrotoxicity EDTA, ethylenediaminetetraacetic acid; INR, international normalized ratio

Time point	Creatinine (mg/dL)	Tacrolimus (ng/mL)	Manganese (µg/L)	INR	Ammonia (µmol/L)
Admission (Day 1)	2.5	5.7	-	-	63.5
Day 2	2.5	-	14.9	1.09	-
Day 10 (pre-EDTA)	2.5	4.6	-	-	-
Day 11 (EDTA, first dose)	2.8	4.4	-	-	-
Day 13 (EDTA, last dose)	3.0	-	1.3	-	-
Day 16	3.5	-	-	-	-
Day 21	2.8	-	0.5	-	-
Day 23	2.7	4.0	-	-	-

**Figure 3:**
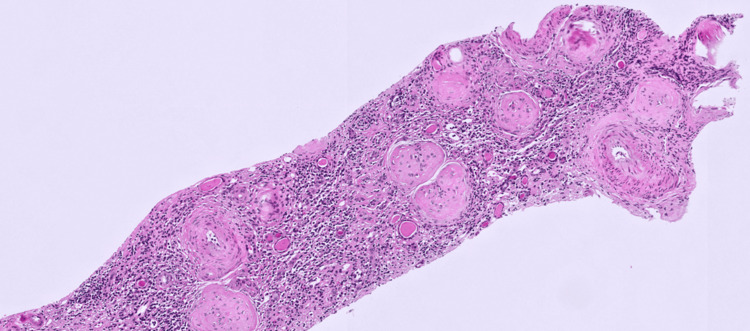
Kidney allograft biopsy, hematoxylin and eosin stain shows significant tubulointerstitial scarring with thickened vessels showing intimal fibrosis

**Figure 4:**
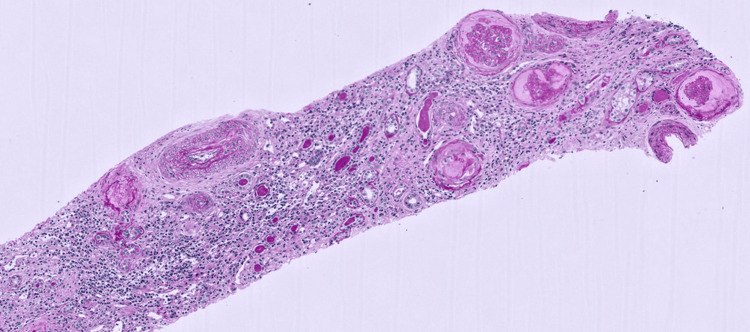
Kidney allograft biopsy, periodic acid-Schiff stain highlighting globally sclerotic glomeruli and extensive interstitial fibrosis with tubular atrophy. Numerous globally sclerotic glomeruli demonstrating an obsolescent pattern of glomerular sclerosis are present, with near-complete cortical scarring

Kidney allograft biopsy, performed approximately one week into the hospital admission due to a rise in serum creatinine, demonstrated severe chronic changes without evidence of acute rejection (Figures 3, 4). Tacrolimus and prednisone were continued, and mycophenolate, which had been held at the time of admission, was resumed once infectious etiologies had been ruled out.

Follow-up and outcomes

Despite early cessation of chelation therapy, Mn levels normalized, and the patient experienced partial clinical improvement. On neurologic follow-up examination in February 2026, she demonstrated intact orientation, coherent thought processes, preserved attention, appropriate recall of personal information, fluent language without dysarthria or dysphonia, and normal mood with good insight and judgment. Formal quantitative neurocognitive assessments were not consistently available.

At follow-up phone call in March 2026, the patient reported persistent but improved neurologic symptoms, including residual tremors, stiffness, and intermittent cognitive difficulties described as "brain fog" and word-finding difficulty. She continued physical and occupational therapy, with significant functional improvement, and remained independent in activities of daily living and instrumental activities of daily living. This aligns with prior reports demonstrating that neurologic manifestations of Mn toxicity may persist despite normalization of serum Mn levels, particularly once advanced disease is established (Table 2) [10,11].

**Table 2:** Timeline of key clinical events Timeline reconstructed from retrospective chart review and authors' interpretation of available records. Timelines are approximate, and clinical details may reflect variability in documentation across providers and institutions over time ADLs, activities of daily living; EGD, esophagogastroduodenoscopy; IADLs, instrumental activities of daily living; MDS/MPN, myelodysplastic/myeloproliferative neoplasm; MRI, magnetic resonance imaging; TIPS, transjugular intrahepatic portosystemic shunt; EDTA, ethylenediaminetetraacetic acid

Year	Event
2013-2014	Recurrent left upper extremity ischemia; left upper extremity amputation
2014	Bone marrow biopsy; diagnosis of JAK2 V617F-positive MDS/MPN
February 2021	Deceased-donor kidney transplantation
March 2021	Presentation with ascites and portal hypertension; liver biopsy showing congestive hepatopathy; Gastroenterology concluded that her presentation was most consistent with Budd-Chiari physiology due to hepatic venous outflow obstruction, potentially related to ongoing thrombotic events in the setting of her underlying JAK2 mutation
April 2021	TIPS placement; EGD demonstrating esophageal varices and portal hypertensive gastropathy
December 2022	Neurology evaluation for "cognitive changes and episodes of loss of time"
2023	Brain MRI for memory decline and episodes concerning for seizures showed increased T1 signal intensity within the globus pallidus and anterior midbrain compatible with manganese related MR signal changes from cirrhosis
June 2023	Neurology follow-up
July 2025	Hospital admission for progressive tremors, gait instability, aphasia, and altered awareness; magnetic resonance imaging demonstrated bilateral T1 hyperintensity of the globus pallidus, consistent with manganese deposition. Subsequently, serum manganese was checked and found to be markedly elevated at 14.9 µg/L
August 2025	Chelation therapy with calcium disodium EDTA (3 days); discontinued due to rising creatinine; kidney allograft biopsy showing chronic changes without acute rejection
March 2026	Six-month follow-up; persistent but improved neurologic symptoms; independent with ADLs/IADLs

## Discussion

Mn is an essential trace element involved in several enzymatic processes, including oxidative metabolism and neurotransmitter synthesis [2]. Under physiological conditions, Mn homeostasis is tightly regulated through hepatobiliary excretion, with minimal contribution from renal clearance [6]. When exposure exceeds the body's capacity for elimination, Mn accumulates within the central nervous system, particularly in the basal ganglia [3,4,6]. Mn crosses the blood-brain barrier via transferrin-mediated transport and divalent metal transporter-1, leading to preferential deposition in the globus pallidus and related structures [4]. This accumulation disrupts dopaminergic neurotransmission and results in manganism, characterized by parkinsonian features, neuropsychiatric symptoms, and gait abnormalities [3,4].

The globus pallidus is a key node in the basal ganglia motor circuit. The direct pathway, facilitated by dopamine via D1 receptors, promotes movement, while the indirect pathway, inhibited by dopamine via D2 receptors, suppresses unwanted movement. Mn disrupts dopaminergic, GABAergic, and glutamatergic neurotransmission across both pathways. This multineurotransmitter disruption accounts for the patient's parkinsonian features, tremor, rigidity, and gait instability, as well as neuropsychiatric manifestations including cognitive difficulties, reflecting involvement of the associative and limbic basal ganglia loops [1].

Clinically, Mn neurotoxicity has been described as a progressive disorder. Early manifestations may include subtle neuropsychiatric symptoms such as irritability, mood changes, insomnia, and cognitive dysfunction. As toxicity progresses, patients may develop neurologic features including tremors, bradykinesia, dystonia, and gait instability. In more advanced stages, a fixed parkinsonian syndrome with postural instability and impaired mobility may develop [10,11]. In contrast to idiopathic Parkinson disease, manganism more frequently presents with early gait disturbance and prominent neuropsychiatric features [3,11].

Neuroimaging plays a key role in supporting the diagnosis. T1-weighted MRI typically demonstrates symmetric hyperintensity within the globus pallidus, reflecting Mn deposition [1]. This finding may be quantified using the pallidal index, which compares signal intensity between the globus pallidus and subcortical white matter [1]. However, this finding is not specific to Mn toxicity and can be seen in other conditions, including hepatic dysfunction and parenteral nutrition. Similarly, serologic testing has significant limitations. Blood Mn level does not always accurately reflect Mn concentration in the targeted tissue, particularly in the brain, and is considered a poor biomarker of Mn exposure or toxicity under many conditions; patients with established neurologic injury may demonstrate normal or only mildly elevated Mn levels despite persistent symptoms [12]. There is currently no gold-standard diagnostic test for Mn neurotoxicity, and diagnosis relies on integrating clinical presentation, exposure history, neuroimaging, and biochemical data [1,12].

Treatment of Mn toxicity primarily involves reducing or eliminating the exposure source. When an identifiable environmental or occupational exposure is present, removal remains the most important intervention [6]. Chelation therapy with calcium disodium EDTA has been used in cases of significant toxicity and has been shown to enhance urinary Mn excretion; however, its effectiveness in reversing established neurologic injury is variable [13]. EDTA therapy has also been associated with nephrotoxicity, particularly in patients with underlying kidney disease, likely related to proximal tubular injury from metal-chelate complexes [13]. Alternative therapeutic approaches have been explored in patients unable to tolerate EDTA. Para-aminosalicylic acid has demonstrated potential benefit in small studies and case reports, possibly through enhanced biliary excretion of Mn [14]. In contrast, dopaminergic therapies commonly used in idiopathic Parkinson's disease have shown limited efficacy in manganism, reflecting differences in underlying pathophysiology [3,11].

The long-term prognosis of Mn neurotoxicity varies depending on the duration and severity of exposure. Although some patients experience partial improvement following a reduction in systemic Mn levels, neurologic deficits often persist due to irreversible injury within basal ganglia structures [10,11]. Early recognition and intervention are, therefore, critical to limiting permanent neurologic impairment.

Mechanistic considerations in portosystemic shunting

The prevalence of Mn-related neuroimaging findings varies by population, with pallidal T1 hyperintensity reported in up to 88% of cirrhotic patients [15]. In patients with portosystemic shunts, including TIPS, Mn accumulation results from two mechanisms: 1) bypass of hepatic first-pass metabolism, allowing intestinally absorbed Mn to enter systemic circulation directly, and 2) impaired hepatobiliary excretion due to underlying liver dysfunction [7,8,16].

Of particular relevance, studies demonstrate that blood Mn concentrations are significantly higher in patients with TIPS compared to those with cirrhosis alone, and TIPS placement can worsen pallidal hyperintensity and neurologic symptoms [15,16]. In the present case, pallidal T1 hyperintensity was identified on brain MRI as early as 2023 and attributed to the patient's history of cirrhosis; her neurologic symptoms at that time were characterized as attentional in nature, and no further neurologic intervention was pursued. This illustrates how pallidal hyperintensity in patients with liver disease and portosystemic shunting may be interpreted as an expected imaging finding, highlighting the importance of considering serum Mn measurement when neurologic symptoms are present.

In hemodialysis patients, increased pallidal signal intensity has been observed despite circulating Mn levels comparable to controls [5,9]. Proposed mechanisms include altered Mn metabolism, increased blood-brain barrier transport, and chronic exposure to trace Mn in dialysis solutions [6,9]. Of note, because circulating Mn is largely protein-bound, hemodialysis is generally ineffective at removing Mn during toxicity [6].

In the present case, the patient's history of Budd-Chiari syndrome with TIPS likely contributed to Mn accumulation through the mechanisms described above. The development of Budd-Chiari syndrome shortly after kidney transplantation, in the context of a known JAK2 V617F mutation associated with hypercoagulability, illustrates the complex interplay between her underlying hematologic disorder and subsequent hepatic venous outflow obstruction. This case demonstrates that a functioning kidney allograft does not protect against Mn toxicity, consistent with the established understanding that systemic Mn homeostasis is maintained primarily through hepatobiliary excretion, while urinary clearance contributes minimally to its elimination [6]. To our knowledge, this represents the first reported case of Mn neurotoxicity occurring in a kidney transplant recipient with a functioning allograft.

Diagnostic pitfalls and therapeutic challenges

This case illustrates several challenges inherent to Mn neurotoxicity. Early symptoms, including cognitive changes, mood disturbances, and insomnia, are nonspecific and easily attributed to other conditions, particularly in patients with complex medical histories. Serum Mn levels are an imperfect biomarker; concentrations may fluctuate and do not reliably reflect brain tissue burden, as levels can normalize despite ongoing neurologic injury [12].

Treatment of Mn toxicity poses additional challenges. Because circulating Mn is largely protein-bound, hemodialysis is ineffective at removing Mn [6]. Chelation therapy with calcium disodium EDTA enhances urinary Mn excretion but has limited efficacy once brain deposition is established [11]. EDTA is also nephrotoxic, which is particularly concerning in transplant recipients with baseline renal impairment [13]. However, chelation therapy is limited by renal function. George et al. reported successful treatment of manganism with kidney transplantation in a dialysis-dependent patient, as restoration of renal function enabled the urinary Mn excretion required for effective chelation [17].

In contrast, our patient developed Mn toxicity despite a functioning allograft, underscoring that renal clearance, while sufficient to facilitate EDTA-mediated excretion, cannot compensate for impaired hepatobiliary elimination in the setting of portosystemic shunting. In patients whose Mn accumulation results from TIPS rather than exogenous exposure, the underlying mechanism cannot be eliminated without compromising portal hypertension management. Finally, the lack of a reliable biomarker to assess tissue burden complicates the monitoring of treatment response.

## Conclusions

Mn-associated neurotoxicity should be considered in transplant recipients presenting with unexplained neurologic symptoms, particularly those with concurrent hepatic dysfunction or portosystemic shunting. Pallidal T1 hyperintensity in patients with liver disease should prompt measurement of serum Mn levels when neurologic symptoms are present. Chelation therapy may be beneficial but requires careful monitoring of kidney function. Early recognition is critical, as neurologic deficits may persist despite normalization of Mn levels once advanced disease is established.
